# Evidence of Renin–Angiotensin System Receptors in Deep Fascia: A Role in Extracellular Matrix Remodeling and Fibrogenesis?

**DOI:** 10.3390/biomedicines10102608

**Published:** 2022-10-17

**Authors:** Carmelo Pirri, Brasilina Caroccia, Andrea Angelini, Lucia Petrelli, Maria Piazza, Carlo Biz, Pietro Ruggieri, Raffaele De Caro, Carla Stecco

**Affiliations:** 1 Department of Neurosciences, Institute of Human Anatomy, University of Padova, 35121 Padova, Italy; 2 Department of Medicine-DIMED, University of Padova, 35128 Padova, Italy; 3 Department of Orthopedics and Orthopedic Oncology, University of Padova, 35128 Padova, Italy

**Keywords:** fascia, thoracolumbar fascia, angiotensin receptor, Ang II type 1 receptor (AT1R), angiotensin-converting enzyme 2 (ACE2), remodeling, renin–angiotensin system, fibrosis

## Abstract

Recent studies have shown that fascial fibroblasts are sensitive to different stimuli (biochemical or biophysical), promoting extracellular matrix remodeling, as well as synthetic activity. Moreover, the extensive literature on the renin–angiotensin system (RAS) reported its involvement in tissue remodeling. This study aimed to investigate the presence of RAS components in the deep fascia. Thoracolumbar fascia specimens were collected from 13 patients (age range: 25–75 years; seven males and five females) who had undergone elective spinal surgical procedures at the Orthopedic Clinic of the University of Padova. Gene expression analysis was performed to investigate the expression of Ang II type 1 receptor (AT1R), Ang II type 2 receptor (AT2R), MAS receptor (MasR), angiotensinogen, angiotensin-converting enzyme 2 (ACE2) and angiotensin-converting enzyme 1 (ACE1). AT1R and ACE2 were also measured with immunoblot. AT1R was the most expressed angiotensin receptor subtype (300.2 ± 317 copies/25 ng of mRNA), followed by MasR (37.1 ± 39.56 copies/25 ng of mRNA) and AT2R (147 ± 122 copies/25 ng of mRNA). The amounts of angiotensinogen, ACE1 and ACE2 were hardly detectable. These findings demonstrate that RAS system receptors are present in the deep fascia, with a greater expression of AT1R, suggesting their involvement in fascial remodeling and fibrogenesis.

## 1. Introduction

Fasciae form a ubiquitous network throughout the whole body and are usually considered a relatively inert tissue with a passive role only in musculoskeletal biomechanics [1]. An increasing number of studies over the last few decades have dedicated quite a bit of attention to fasciae, showing the role of muscular fascia in pain, proprioception, motor coordination and biomechanical behavior [2,3,4].

It is now well-known that deep fascia is a well-defined fibrous sheath that covers and keeps in place a group of muscles (aponeurotic fascia), or a single muscle (epimysial fascia), or serves for the insertion [5]. Moreover, Bhattacharya et al. [6] described a subfascial and suprafascial vascular network connected by extensive vascular arcades traversing the fascial planes. The deep/muscular fascia contains well-defined lymphatic vessels with a high rate of flow of lymphatic fluid [7], different kinds of cells embedded in the extracellular matrix (ECM), and numerous nerve fibers [8]. The predominant cell population found in fascial tissue are fibroblasts [9,10]; their main role is to maintain tissue integrity and organization, as they are involved in mechanotransduction and in secreting precursors of the extracellular matrix. The fascial ECM is composed of a tridimensional, highly dynamic matrix, made up of collagens, proteoglycans/glycosaminoglycans, elastin, fibronectin, laminins, and several other glycoproteins. Together, these components comprise a complex network in which the cells reside, communicate, and are involved in various cellular functions [5,11]. Although recent studies showed that fascial fibroblasts are sensitive to different stimuli, from biochemical [12,13] to biophysical [14], promoting ECM remodeling, as well as synthetic activity (collagen fibers of type I and III, and hyaluronan). Stecco et al. [15] suggested that fibrosis may occur in different organs as a long-term consequence of hyaluronan densification due to excessive collagen fiber deposition and consequent ECM remodeling, and this mechanism was also found in the deep fascia [15].

There is extensive literature on the renin–angiotensin system (RAS) involvement in the progression of fibrosis [16,17,18,19,20]. The RAS is a peptidergic system with endocrine features, that exists in the circulation and tissues such as the heart, vasculature, skin, eye and nervous system. Angiotensin II (Ang II) is the main biologically active element of RAS, which has an important role in regulating blood pressure. Ang II is generated by the proteolytic conversion of angiotensinogen in the decapeptide Angiotensin I (Ang I), then Ang I is converted to the octapeptide Ang II by the angiotensin-converting enzyme (ACE1). Moreover, Ang II has been reported to be associated with increased fibrosis in many tissues, such as the liver [21], kidney [17], cardiac muscles [22], skin [23], eye [24] and other tissues by enhancing ECM protein synthesis, such as fibronectin (FN), collagens, etc., through the Ang II type 1 receptor (AT1R). Ang II mainly exerts its function through two receptor subtypes, the AT_1_R and type 2 receptor (AT2R). Of note, proteolytic fragments of Ang II, such as Angiotensin 1-7, formed by the proteolytic action of angiotensin-converting enzyme 2 (ACE2), also have biological activity via the MAS receptor. It is currently accepted that two different branches of the RAS exist: the harmful one, ACE–Ang II-AT1R, and the beneficial one ACE2–Ang 1-7-MasR axis, which is known to counterbalance the former [25].

Besides a systemic RAS playing an endocrine role in the human body, the concept of a local RAS was also demonstrated, having autocrine, paracrine and intracrine functions. Indeed, even locally, RAS components can be produced and be active, limited to tissues or organs such as the kidney, heart, vessels, adipose tissue, lymphatic tissue, and adrenal glands [26,27,28].

Therefore, the current study aimed to investigate the expression of Ang II receptors in the deep fascia as their presence may better explain fascial ECM remodeling and the role of Ang II in the dysfunctions relating to the deep fascia. To achieve this goal, the thoracolumbar fascia (TLF) was characterized and the presence of AT1R, AT2R and MAS receptor (MasR) was investigated with digital droplet PCR. Angiotensinogen (AGT), ACE2 and ACE1 expression were also measured to clarify if angiotensin peptides can be locally generated and functionally act in this tissue.

## 2. Materials and Methods

### 2.1. Patients and Tissues

**Figure 1:**
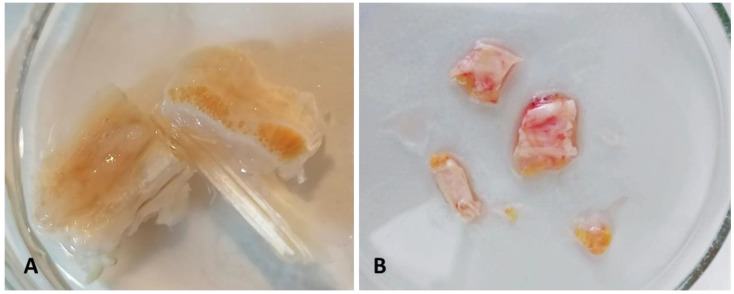
(**A**) 1 cm × 1 cm samples for histological and immunohistochemistry examinations after isolation were immediately fixed in formalin. (**B**) 1 cm × 1 cm TL fasciae were cut into small pieces, immediately snap-frozen in isopentane on dry ice, and then stored in liquid nitrogen in the institutional tumor bank for total ribonucleic acid (RNA) extraction, gene expression analysis and immunoblotting.

We obtained TLF specimens under sterile conditions in the operating room from 13 informed and consenting patients (age range: 25–75 years; seven males and five females) who were undergoing elective spine surgical procedures at the Orthopedic Clinic of the University of Padova. Exclusion criteria were malignant neoplasms, previous spine surgery, acute inflammatory disease, infectious diseases, high blood pressure or cardiovascular diseases. All the samples were at least 2 cm × 2 cm, and were divided 1 cm × 1 cm for histological and immunohistochemistry examinations, after isolation they were immediately fixed in formalin (Figure 1A), in confirmation of having taken TLF without vessels; the other 1 cm × 1cm deep fasciae were cut into small pieces (Figure 1B), immediately snap-frozen and then stored in liquid nitrogen until total ribonucleic acid (RNA) extraction, gene expression analysis and immunoblotting were performed. The collection in the institutional tumor bank and the use of the fascia tissues were approved by the Ethics Committee of the University Hospital of Padova (approval no. 3722/AO/16).

### 2.2. Histological Staining

The 13 full-thickness TL fasciae specimens were immediately mounted on cardboard to avoid deformation artifacts, fixed in 10% formalin solution and embedded in paraffin. Five-µm-thick sections were stained with hematoxylin and eosin, and Alcian Blue.

### 2.3. Immunocytochemistry to Detect Collagen Type I and III

TL fasciae sections were immunolabeled with goat anti-collagen-type I or rabbit anti-collagen type III antibodies. Briefly, endogenous peroxidases were inactivated with PBS + 0.5% H_2_O_2_ for 10 min at room temperature; slides were then preincubated with blocking buffer (0.1% BSA in PBS) for 1 h at room temperature to decrease background staining. Sections were then incubated overnight at 4 °C with anti-collagen type I (1:400, SouthernBiotech, Birmingham, AL, USA) or anti-collagen type III (1:100, Abcam, Cambridge, UK) antibodies after washing with PBS; antigens were detected by incubation with secondary antibodies labeled with horseradish peroxidase (HRP) and 3,3**′**–diaminobenzidine (Liquid DAB plus substrate Chromogen System kit; Dako, Santa Clara, CA, USA). Negative controls were performed by the omission of the primary antibody, confirming the specificity of the immunostaining.

### 2.4. Total Ribonucleic Acid (RNA) Extraction

Total RNA extraction from 13 deep fascia samples was performed using TRIzol**^®^** reagent according to the manufacturer’s instructions. 100 mg of deep fascia was ground into a fine powder in a refrigerated and sterilized pestle and mortar; then 1 mL of TRIzol**^®^** reagent was added. The mixture was incubated at room temperature for 5 min; Then, 0.3 mL of prechilled chloroform was slowly added to the microcentrifuge tube and mixed gently for 30 s. Samples were centrifuged at 12,000× *g*, 4 °C for 15 min, and the supernatants were transferred to a new tube. Total RNA was obtained through precipitation with an equal volume of chilled isopropanol at −80 °C for 30 min. The tubes were then centrifuged at 12,000 rpm for 10 min and supernatants were carefully discarded. The pellets were washed once with 75% ethanol and air-dried. Total RNA was dissolved in 50 µL of nuclease-free water and stored at −80 °C. Sample concentration and purity were determined using a NanoDrop 1000 Spectrophotometer (Thermo Scientific, Milan, IT, USA).

### 2.5. Gene Expression Analysis

We measured the absolute gene expression of *AGT*, *AT1R*, *AT2R* and *MasR*, *ACE1,* and *ACE2* mRNA with a digital droplet PCR system (ddPCR) (Bio-Rad QX200TM, Bio-Rad Laboratories, Segrate, Milano, IT). One µg total RNA was reverse-transcribed with Iscript (Bio-Rad, Milan, IT) in a final volume of 20 µL and 25 ng of RNA was analyzed in ddPCR. The reaction mix was prepared with QX200 ddPCR EvaGreen supermix (Bio-Rad Laboratories, Segrate, IT) and the specific primer at a final concentration of 300 nM. Droplets were generated using the QX200™ Automated Droplet Generator (Bio-Rad Laboratories, Segrate, IT) and then transferred to a 96-well PCR plate for amplification in the Bio-Rad C1000™ (Bio-Rad Laboratories, Segrate, IT). After PCR, droplets from each sample were acquired in a QX100 Droplet Reader (Bio-Rad Laboratories, Segrate, IT) and the obtained data were analyzed with QuantaSoft analysis software (Bio-Rad Laboratories, Segrate, IT). Absolute levels of the target gene were expressed as numbers of copies for 25 ng of RNA. Primers for the amplification of the genes of interest were designed using Primer3web online software and are reported in Table 1.

### 2.6. Immunoblotting

Immunoblotting for AT1R and ACE2 was performed following a standard protocol. Protein samples were obtained from eight samples of deep fasciae. Briefly, TLF fasciae were homogenized in RIPA Lysis Buffer (Thermo Scientific, Milan, IT) and protein concentration was measured with a BCA Protein Assay Kit (Thermo Scientific, Milan, IT). Lysate fraction (50 µg) was separated in a polyacrylamide gel and electroblotted onto a nitrocellulose membrane (Amersham-Hybond EC, GE Healthcare Life Sciences, Milan, IT). The membrane was blocked for 30 min at room temperature in 5% non-fat dry blocking milk and then incubated overnight at 4 °C with a primary antibody against AT1R (diluted 1:1000, Abgent, San Diego, CA, USA) or ACE2 (diluted 1:500, Abcam, Cambridge, UK). After washing, the membrane was incubated for 1 h with a peroxide-linked secondary antibody and revealed through a chemiluminescent reaction. Then, band intensity was measured with a VersaDoc Imaging System (Bio-Rad Laboratories, Segrate, IT).

### 2.7. Statistical Analysis

Statistical analysis was performed using GraphPad 8.4.2. (GraphPad Software Inc., San Diego, CA, USA), and a *p <* 0.05 was always considered as a limit for statistical significance. The normality assessment was carried out using the Kolgomorov–Smirnov test. All results are presented as the mean ± standard deviation. Receptor data were analyzed using one-way analysis of variance (ANOVA) followed by Tukey’s test for multiple comparisons. Differences between ACE1 and ACE2 enzymes were analyzed by paired *t*-test.

## 3. Results

### 3.1. TL Fasciae Histological Description

**Figure 2:**
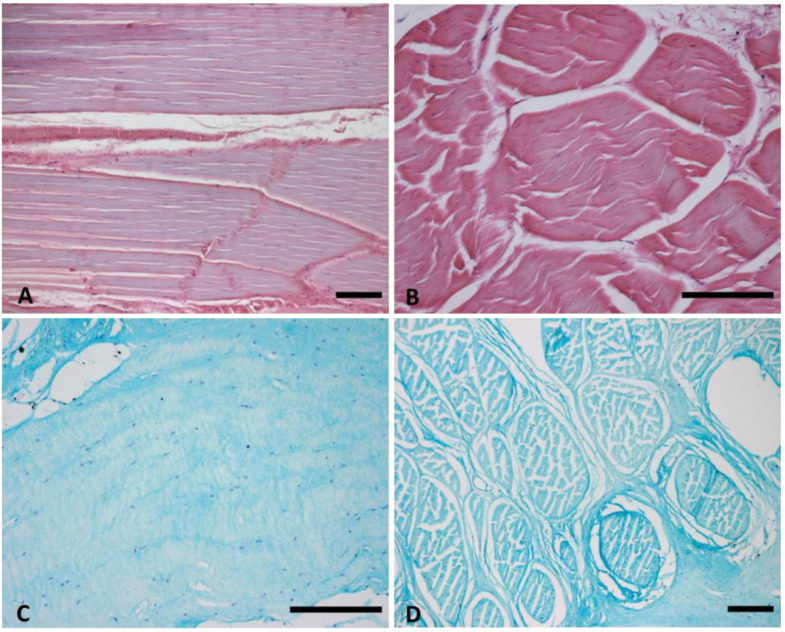
Haematoxylin and eosin (**A**,**B**) and Alcian Blue (**C**,**D**) staining in longitudinal (**A**,**C**) and transversal (**B**,**D**) directions. Scale Bars: 200 µm. Magnification: (**A**,**D**) = 5×; (**B**,**C**) = 10×.

**Figure 3:**
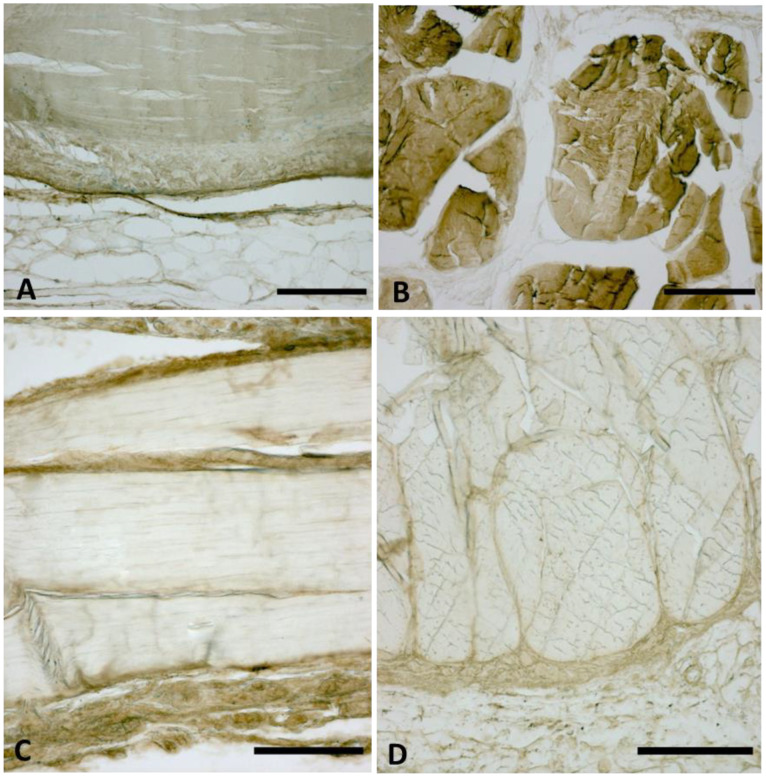
Immunocytochemistry to detect collagen type I (**A**,**B**) and collagen type III (**C**,**D**)*,* in longitudinal (**A**,**C**) and transversal (**B**,**D**) directions. Scale Bars: 200 µm. Magnification: (**A**–**D**) = 10×.

The histological staining and the collagen types I and III immunolabeling confirmed the exact sampling of TLF without blood vessels. Qualitatively, the TL fasciae showed the dual orientation of connective fibers (longitudinal and transversal) and the histological features with an important fibrous component and proteoglycans/glycosaminoglycans component (Figure 2 and Figure 3).

### 3.2. AT1R, AT2R and MasR Expression in Deep Fascia

Angiotensin receptors expression, i.e., *AT1R*, *AT2R* and *MasR*, in 13 samples of deep fascia was measured with ddPCR. We found that all receptors were expressed in the thoracolumbar fascia (TLF), albeit in varying amounts (Table 2). *AT1R* was the most expressed angiotensin receptor subtype, even if it showed high variability among the different samples analyzed. Of note, *AT1R* expression was detectable in 11 samples analyzed, *AT2R* was measured in 8 subjects and *MasR* was measured in only 10 samples. In detail, *AT1R* levels were 5 and 1.5 times higher than *AT2R* and *MasR*, respectively.

**Figure 4:**
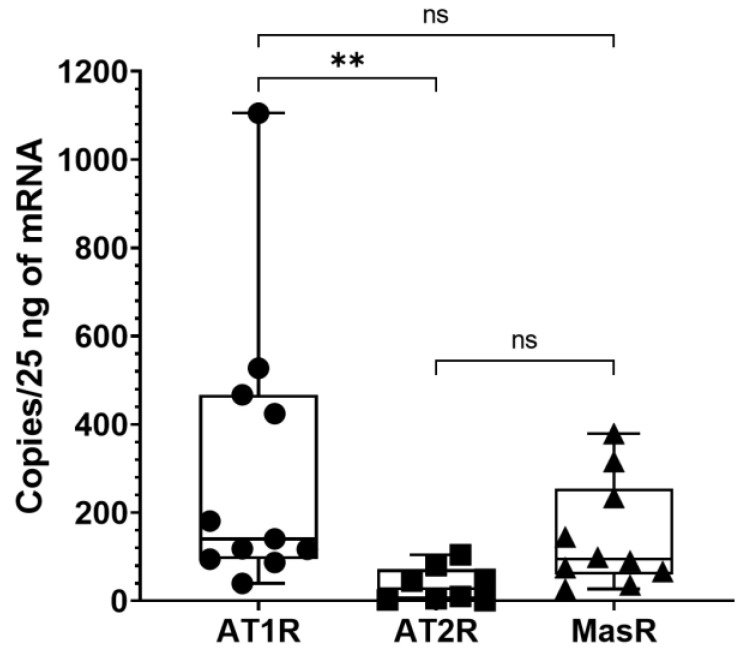
Gene expression of angiotensin receptors in human thoracolumbar fascia. The amount of the different angiotensin receptors (*AT1R, AT2R, MasR*) in thoracolumbar fascia was measured with ddPCR. The rank of expression of angiotensin receptors was *AT1R* > *MasR* > *AT2R*. In the table are reported the values obtained from ddPCR. Data are expressed as gene copies obtained from 25 ng of mRNA. **: *p* < 0.01; n.s.: not significant.

According to Tukey’s multiple comparisons tests, the comparison between the different receptors showed a statistically significant difference for *AT1R* vs. *AT2R* (*p* < 0.01), whereas it did not show a statistically significant difference for *AT1R* vs. *MasR* (*p* > 0.05) and for *AT2R* vs. *MasR* (*p* > 0.05) (Figure 4).

**Figure 5:**
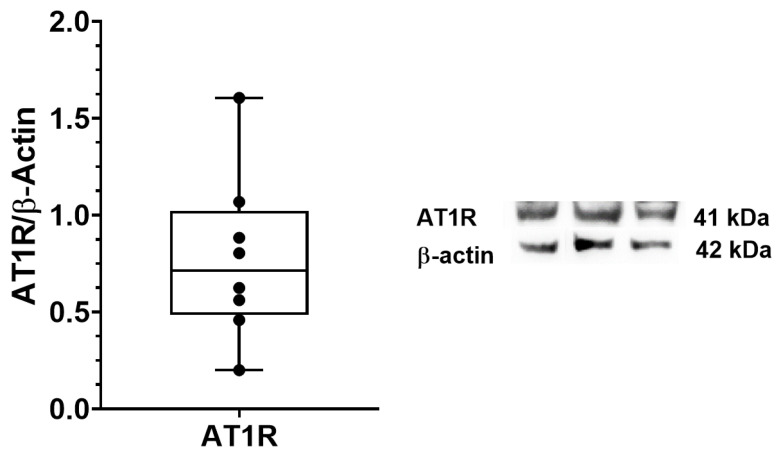
AT1R protein expression in human thoracolumbar fascia. Immunoblotting of AT1R protein in TLF (n = 8). Relative quantitation of AT1R vs. β-actin expression is shown in the bar graph.

*AT1R* expression in TLF was also investigated with immunoblotting in eight samples. We confirmed the expression of AT1R at protein levels in deep fascia (Figure 5).

Moreover, to clarify if Ang II and Ang 1-7, the two biologically active peptides, could be locally produced, we measured the expression of AGT, *ACE1* and *ACE2* in TLF tissue. Of note, the data showed that angiotensinogen was undetectable in all examined tissues, instead, *ACE1* and *ACE2* were hardly detectable (Table 3), *ACE1*: 3.38 ± 2.86 copies/25 ng of mRNA; *ACE2*: 0.77 ± 0.36 copies/25 ng of mRNA, respectively.

**Figure 6:**
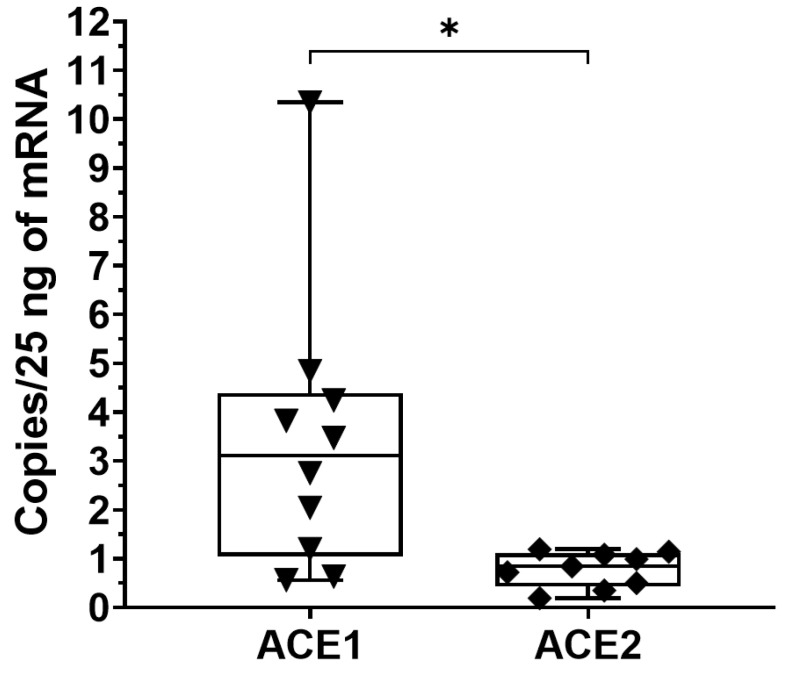
Gene expression of *ACE2* and *ACE1* in human thoracolumbar fascia. *ACE1* and *ACE2* expressions were investigated with ddPCR. Both enzymes were expressed at low levels (*ACE1*: 3.38 ± 2.86 copies/25 ng of mRNA; *ACE2*: 0.77 ± 0.36 copies/25 ng of mRNA, respectively); *: *p* < 0.05.

According to the paired *t*-test, the comparison of *ACE1* vs. *ACE2* showed a statistically significant difference (*p* < 0.05) (Figure 6).

Considering the low levels of ACE2 gene expression, we investigated its expression in five samples at protein levels and no bands were detected with immunoblot, suggesting that ACE2 is not present in TLF and unlikely to be making Ang 1-7 in local production.

## 4. Discussion

**Figure 7:**
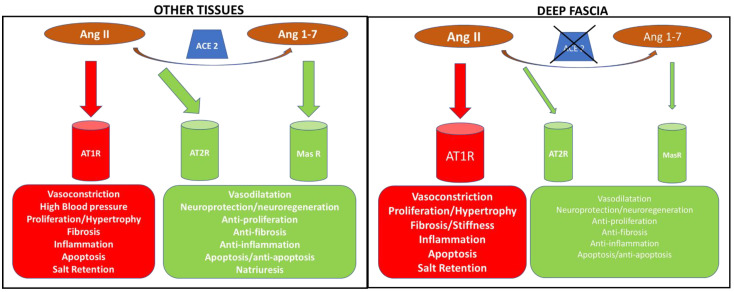
Schematic representation of the RAS system in tissues (**left** panel) and deep fascia (**right** panel), underlining the different effects exerted by angiotensin via RAS receptors, as reported in the literature for the other tissues and as we hypothesized in deep fascia. The Ang II effects via AT1R on the deep fascia are under investigation. Ang II: angiotensin II; Ang 1-7: angiotensin 1-7; AT1R: AngII type 1 receptor; AT2R: Ang II type 2 receptor; MasR: Mas receptor; ACE2: angiotensin-converting enzyme 2; ACE1: angiotensin-converting enzyme 1.

This is the first time that renin–angiotensin system components have been described in human TLF. The expression of angiotensin receptors, such as AT1R, AT2R and MasR, were investigated with digital droplet PCR: *AT1R* was the most expressed receptor subtype (300.2 ± 317 copies/25 ng of mRNA), followed by MasR (37.1 ± 39.56 copies/25 ng of mRNA) and AT2R (147 ± 122 copies/25 ng of mRNA). Moreover, RAS proteolytic enzyme expression was also investigated, i.e., *ACE1* and *ACE2,* and found that they were hardly detectable, the levels being 3.38 ± 2.86 copies/25 ng of mRNA and 0.77 ± 0.36 copies/25 ng of mRNA, respectively. These data are of much interest because the presence of angiotensin receptors in the fascial tissue, mainly AT1R, suggests a possible role in the fascial remodeling and fibrogenesis of Ang II. Of note, Ang II is the main component of the RAS, extensively studied and reported to promote the fibrotic process in many tissues [21,22,23,24]. The RAS is a complex and dynamic two-axis molecular cascade present in different organ systems, which plays a crucial role in their homeostasis [25]. The classical axis including ACE1, Ang II and AT1R, mediates the body fluid homeostasis, vasoconstriction, fibrosis, inflammation, cellular growth, migration, cardiac hypertrophy, thrombosis, and reactive oxygen species (ROS) production [25]. A counter-regulatory axis is formed by ACE2, Angiotensin 1-7 (Ang 1-7) and the MasR [25]. Ang 1-7, acting via MasR, exerts anti-inflammatory, antifibrotic, antiproliferative, antioxidative, vasodilator and antithrombotic effects [26]. Moreover, Ang II can also act through the AT2R with effects similar to that of MasR [29]. ACE2 degrades Ang II to Ang 1-7, thus reducing Ang II effects in vasoconstriction, fibrosis and sodium retention [29] (Figure 7). 

Ang II-induced fibrosis is mainly mediated by AT1R, as it was attenuated when AT1R was silenced in fibroblasts [30]. Conversely, when the human AT1R transgene was expressed in a mouse model, progressive cardiac remodeling was induced with the proliferation and activation of cardiac fibroblasts [31].

The comparison of RAS receptors expression in deep fascia showed a statistically significant difference for *AT1R* vs. *AT2R* (*p* < 0.01), whereas it did not show a statistically significant difference for *AT1R* vs. *MasR* (*p* > 0.05) and for *AT2R* vs. *MasR* (*p* > 0.05). These results provide compelling evidence for a preponderant role of AT1R in deep fasciae, through which the circulating Ang II could perform its functions as in the other tissues (Figure 7). An in-depth analysis of biochemical features is a task for future investigations. Collagens type I and III and hyaluronan (HA) are key components that guarantee force transmission [32] and fascial gliding with respect to the all-around structures [5]. The increase in the secreted HA can cause, if temporary, fluidity of the tissue, facilitating fascial gliding [14]. Pavan et al. [33] reported that increased stiffness of the ECM is mainly caused by collagen accumulation as with human skeletal muscle in their fasciae. Collagens type I and III are two key elements in fascial remodeling [14]; both are the main constitutive elements of the ECM fascial fibrous component, together with HA which participates with the ECM fascial aqueous matrix component [14]. Ang II through AT1R could promote the fascial remodeling of these elements and fibrinogenesis up to fibrosis. Indeed, the latter, as reported by Stecco et al., in some organs and tissues as fasciae, may occur as a direct consequence of HA densification in the long term [15]. Moreover, Schleip et al. [1] observed that the density of fascial myofibroblasts (MFBs) in human lumbar fascia could be associated with an augmented occurrence of (micro)injuries and related repair processes in the TLF. Fibrogenesis is a mechanism of wound healing and repair and prolonged injury causes the deregulation of normal processes resulting in the extensive deposition of ECM proteins and fibrosis [34]. MFB accumulation and excessive deposition of ECM components are common features at the stage of fibrosis. Ang II modulates the profibrotic and vasoconstrictors' actions through the AT1R expression on MFBs in the heart and kidney [35,36]. The fascial MFBs, known for their important role in some pathological fibrotic contractures that affect the fasciae, could be modulated in their actions by Ang II via AT1R.

Our data also confirmed the presence in deep fascia of counter-regulatory axes (AT2R and MasR), which could guarantee, as in the other tissues, reductions in Ang II effects [25]. Finally, we found that the *ACE1* and *ACE2* were hardly detectable, *ACE1*: 3.38 ± 2.86 copies/25 ng of mRNA, *ACE2*: 0.77 ± 0.36 copies/25 ng of mRNA, with a statistically significant difference of *ACE1* vs. *ACE2* (*p* < 0.05). Considering the low levels of *ACE2* gene expression, we investigated if ACE2 protein was present in the TLF and we found no expression of ACE2 with immunoblot. Of note, we also investigated the expression of AGT in the TLF and it was undetectable in all samples examined. Consequently, our results indicate the absence, in the deep fascia, of a local RAS able to produce *in-site* Ang II and to convert the circulating Ang II in Ang 1-7. We can mainly speculate that fascial remodeling by AT1R is due to circulating Ang II, which could be able to stimulate fibrogenesis in the deep fasciae.

However, some limitations are worth noting. This is the first study that investigated the RAS receptors' expression in deep fascia; a more complete assessment of deep fasciae collected from different topographical districts of the human body would give an even more complete view. Moreover, an in-depth analysis of biochemical features should be a task for future investigations. Nonetheless, this work constitutes the first step toward understanding the RAS system's role in fascial remodeling and fibrogenesis.

## 5. Conclusions

These findings demonstrate that RAS receptors are present in the TLF, a deep fascia, with greater expression of AT1R, suggesting their involvement in fascial remodeling and fibrogenesis. This study indicates that Ang II, by AT1R, might be identified as a risk factor for fascial fibrosis. We are sure that by integrating research efforts we will better understand the role of RAS in the fascial tissue, identifying the components targetable by drugs and treatments.

## Figures and Tables

**Table 1:** Primer sequence used in the digital droplet PCR assay.

	Forward	Reverse
AGT	TCCAGCCTCACTATGCCTCT	GCGGTCATTGCTCAATTTTT
AT1R	ATGATTCCAGCGCCTGAC	GGTCCAGACGTCCTGTCACT
AT2R	GGTTTCTAGCATATACATCTTCAACCT	TTGCCCATAGAGGAAGAGTAGC
MasR	TTCGCTATGCCCATGAGACT	TGGTGTAGGTTCCCAAAGGT
ACE1	AGGAGCAGAACCAGCAGAAC	TCAGCCTCATCAGTCACCAG
ACE2	AAAGTGGTGGGAGATGAAGC	GAGATGCGGGGTCACAGTAT

**Table 2:** *AT1R*, *AT2R* and *MasR* values obtained from ddPCR. Data are expressed as gene copies obtained from 25 ng of mRNA.

	AT1R	AT2R	MasR
Number of values	11	8	10
Minimum	39.77	0.63	26.88
25% Percentile	94.8	2.74	59.16
Median	140.6	27.59	95.09
75% Percentile	466.9	72.45	254.7
Maximum	1105	104.8	379.3
Range	1065	104.1	352.4
Mean	300.2	37.09	147
Std. Deviation	317	39.56	121.9
Std. Error of Mean	95.57	13.99	38.55

**Table 3:** *ACE1* and *ACE2* values obtained from ddPCR. Data are expressed as gene copies obtained from 25 ng of mRNA.

	ACE1	ACE2
Number of values	10	9
Minimum	0.56	0.19
25% Percentile	1.05	0.42
Median	3.11	0.84
75% Percentile	4.39	1.11
Maximum	10.34	1.19
Range	9.78	1
Mean	3.38	0.77
Std. Deviation	2.86	0.36
Std. Error of Mean	0.90	0.12

## Data Availability

The data presented in this study are available on request from the corresponding author. The data are not publicly available due to privacy.
